# Novel immunotherapies for adult patients with B-lineage acute lymphoblastic leukemia

**DOI:** 10.1186/s13045-017-0516-x

**Published:** 2017-08-18

**Authors:** Guoqing Wei, Jiasheng Wang, He Huang, Yanmin Zhao

**Affiliations:** Bone Marrow Transplantation Center, The First Affiliated Hospital, School of Medicine, Zhejiang University, Hangzhou, 310000 China

## Abstract

The past decade witnessed the rapid development of adult B-lineage acute lymphoblastic leukemia (ALL) treatment. Beyond the development of chemotherapy regimens, immunotherapy is starting a new era with unprecedented complete remission (CR) rate. Targeting B-lineage-specific surface markers such as CD19, CD20, CD22, or CD52, immunotherapy has been demonstrating promising clinical results. Among the immunotherapeutic methods, naked monoclonal antibodies (mAbs), antibody-drug conjugate (ADC), bispecific T cell engager (BiTE), and chimeric antigen receptor (CAR) T cells are the main types. In this review, we will examine the emerging preclinical and clinical development on (1) anti-CD20 naked mAbs rituximab, ofatumumab, and obinutuzumab; (2) anti-CD19 ADCs SAR3419 and SGN-CD19A and anti-CD19 BiTE blinatumomab; (3) anti-CD22 naked mAb epratuzumab and anti-CD22 ADC inotuzumab ozogamicin; (4) anti-CD52 naked mAb alemtuzumab; and (5) anti-CD19 CAR T cells. We will discuss their efficacy, adverse effects, as well as future development.

## Background

For children with B cell acute lymphoblastic leukemia (B ALL), current chemotherapy regimens can achieve long-term overall survival (OS) of 80–90%. However, similar results have not been seen in adults. Despite a high initial complete response (CR) rate of 80–90%, most of the adults will eventually relapse with chemotherapy-resistant disease. Long-term OS in adults with B ALL remains in the range of 30–50%; the prognosis of relapsed or refractory (R/R) ALL is even more dismal with a 5-year OS of only 10% [1, 2]. For R/R ALL patients, the only option to achieve long-term survival is allogeneic hematopoietic stem cell transplantation (allo-HSCT), which requires reinduction chemotherapy prior to the transplantation. The chemotherapy in the context, however, is generally poorly tolerated with unsatisfied outcomes, as only 5 to 10% patients can be bridged to allo-HSCT [3]. Although a few new cytotoxic drugs have been approved over the last decade such as clofarabine and liposomal vincristine, the low single-agent response rates (17% with clofarabine monotherapy, 20% with liposomal vincristine monotherapy) still emphasize an urgent need for different alternative treatment strategies in R/R adult ALL [4, 5].

**Fig. 1:**
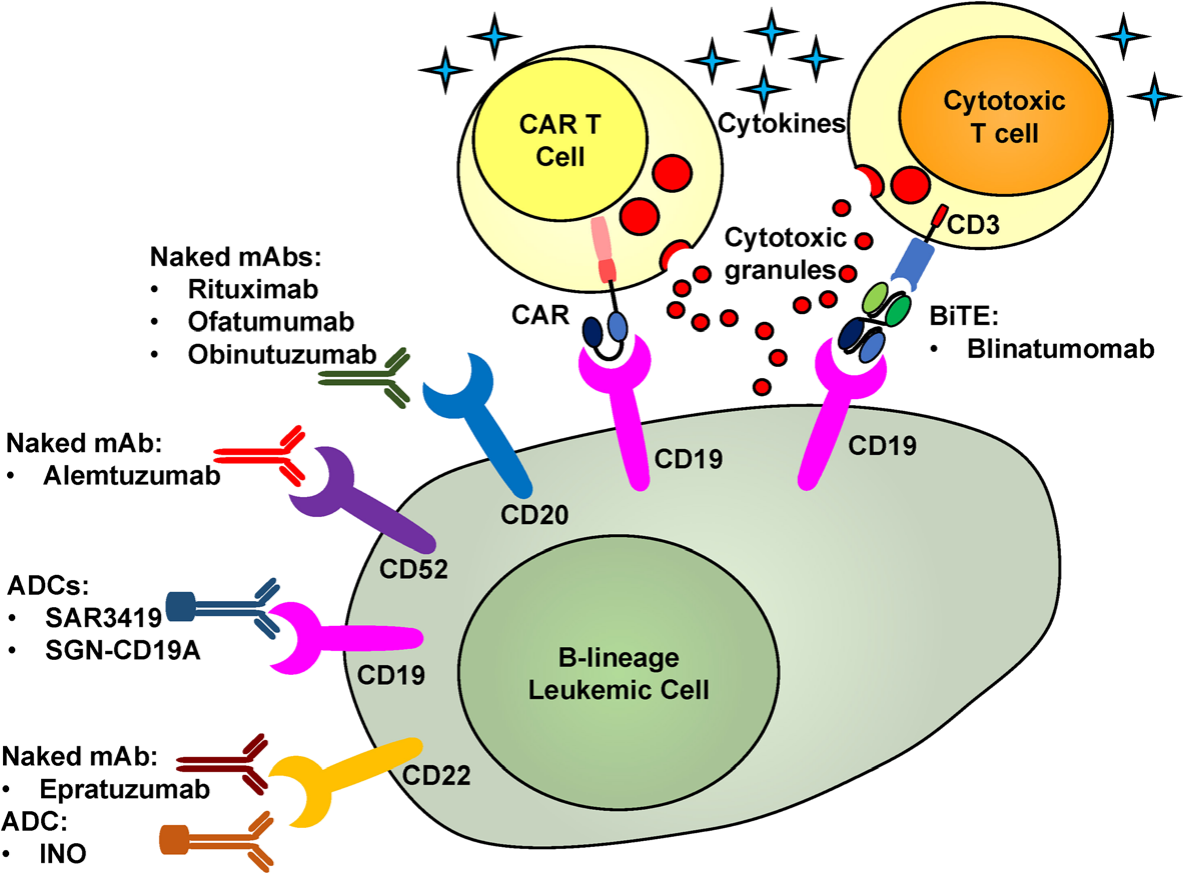
Different mechanisms of immunotherapies treating ALL

Altogether, four types of immunotherapies have been developed to date, including naked monoclonal antibodies (mAbs) (such as rituximab, epratuzumab, and alemtuzumab), conjugated monoclonal antibodies (such as inotuzumab ozogamicin, SAR3419, and SGN-CD19A), bispecific T cell engager (BiTE) (such as blinatumomab), and chimeric antigen receptor (CAR) T cell therapy (Fig. 1). Naked monoclonal antibodies exert their cytotoxic effects through mechanisms such as antibody-dependent cytotoxicity, complement-dependent cytotoxicity, and direct induction of apoptosis; moreover, direct blocking of leukemic cell receptors can lead to cell death if the signalings through the receptors are crucial for leukemic cell to survive. If a surface marker is known to internalize upon binding (such as CD19 and CD22), potent cytotoxins can be conjugated to the monoclonal antibody, resulting in an additional cytotoxic mechanism. BiTE conjugates two monoclonal antibodies recognizing leukemic cell and cytotoxic T cells (CTLs) and exerts its effects by specifically bridging CTLs and leukemic cells. CAR T cells utilizes engineered T cells by introducing leukemic cell-targeting single-chain variant fragment (scFv) chimerized with intracellular T cell activation domains. Both BiTE and CAR T cells lead to leukemic cell killing in mechanisms similar to cancer-specific CTLs, including releasing of cytotoxic granules, activation of death-related receptors, and releasing of cytokines. Compared with CAR T cell therapy, naked/conjugated mAbs and BiTE are more readily available and easier to manufacture; however, CAR T cell therapy as a “living drug” is more durable and repeat infusions are usually not needed. Based on clinical data, BiTE and CAR T cell therapy are more potent and generate better outcomes than naked/conjugated mAbs; however, these two modalities are associated with more severe side effects, such as cytokine release syndrome (CRS), and adverse neurologic events. In this review, we will discuss clinical and pre-clinical results of these different modalities in treating B ALL, focusing on the efficacy (Table 1) and the side effects (Table 2).

**Table 1:** Major clinical trials on monoclonal antibodies treating acute lymphoblastic leukemia

Targets	Medications	Patients	Regimens	Outcome	Study
CD20	Rituximab	216 pts with de novo Ph-negative B ALL; median age was 46 years (range, 16–84)	Combination of hyper-CVAD plus rituximab	CRD and OS were better with the combination of hyper-CVAD plus rituximab than with hyper-CVAD alone (69 vs 38%; *P* < .001 and 71 vs 47%, *P* = .003) for the younger pts (age < 60 years)	Phase III [8]
		220 pts aged 18–59 years old with newly diagnosed CD20-positive Ph-negative B cell precursor (BCP) ALL	Rituximab (375 mg/m^2^) was added to pediatric-inspired GRAALL protocol from induction to the first year of maintenance for a total of 16 to 18 infusions	After induction ± salvage reinduction, CR rate was 92 and 91% in the rituximab and control arm. Pts treated in the rituximab arm had a lower CIR (2-year CIR, 18 vs 30.5% in the control arm; *p* = 0.02) and longer EFS (2-year EFS, 65 vs 52% in the control arm; *p* = 0.038), but not longer OS (2-year OS, 71 vs 64% in the control arm; *p* = 0.095)	Phase III [9]
	Ofatumumab	55 pts with de novo ALL and 4 pts in CR previously treated; median age was 41 years (18–71)	Hyper-CVAD in combination with ofatumumab Ofatumumab given on courses 1 and 3, and 4 courses of MTX-Ara-C	98% CR rate after cycle 1, 53 (93%) pts achieved MRD negativity. The 3-year CRD and OS rates were 78 and 68%, respectively	Phase II [13]
CD19	Blinatumomab	116 pts with Ph-negative BCP ALL with hematologic CR and MRD ≥ 10^− 3^ after ≥ 3 intensive chemotherapy treatments; Median age was 45 years (18–76)	4-week continuous IV infusion, followed by a 2-week break (1 cycle). MRD responders in cycle 1 received up to 3 additional cycles or underwent HSCT	Complete MRD response after the first cycle was 78%; complete MRD response rate was 80%. Median OS and RFS were 18.9 and 36.5 months, respectively	Phase II [26]
		36 pts with R/R pre-B ALL; median age was 32 years (18–77)	4-week continuous infusion followed by a 2-week interval	69% hematologic response and 88% of the responders also obtaining a molecular response (MRD level below 10^− 4^ by PCR) within the first 2 cycles	Phase II [27]
CD22	Epratuzumab	30 pts with R/R CD22+ B ALL; median age was 35 years (21–59)	360 mg/m^2^/day on days 1, 8, 15, and 22, combined with hyper-CVAD	The ORR was 50% including 9 CR (30%), 1 CRi (3%), and 5 PR (17%). All pts have died (during aplasia *n* = 3, progression *n* = 23, multiple organ failure *n* = 1), except the 3 responders still in CR, but yet recently enrolled	Phase II [37]
		31 pts with R/R Ph-negative B ALL. Median age was 41 years (21–69)	Clofarabine 40 mg/m^2^/day on days 2–6, cytarabine 1 g/m^2^/day on days 1–5, epratuzumab 360 mg/m^2^/day on days 7, 14, 21, and 28	10 pts achieved CR and 6 achieved CRi for a CR/CRi rate of 52%. The median OS was 5 months	Phase II [38]
	Inotuzumab ozogamicin	90 pts with R/R pre-B ALL; median age was 39.5 years (range 4–84)	INO single-dose at 1.8 mg/m^2^ every 3–4 weeks, *n* = 49; INO weekly at 0.8 mg/m^2^ on day 1 and at 0.5 mg/m^2^ on days 8 and 15, every 3 to 4 weeks, *n* = 41	17 pts (19%) CR, 27 (30%) CRp, and 8 (9%) marrow CR (no recovery of counts). ORR was 58%. Response rates were similar single dose and weekly dose (57 vs 59%). The median survival was 6.2 months: 5.0 months with single dose and 7.3 months with weekly dose	Phase II [40]
		326 CD22-positive, R/R ALL pts underwent randomization, the first 218 (109 in each group) were included in the analysis of complete remission	INO group: INO (0.8–0.5 mg/m^2^, weekly, 3 times per cycle; cycle length, 21–28 days; total number of cycles, 6); standard intensive chemotherapy: FLAG for up to 4 cycles, cytarabine plus mitoxantrone for up to 4 cycles, or high-dose cytarabine for up to 1 cycle	CR rate was higher with INO than with standard therapy (80.7 vs. 29.4% *p* < 0.001) and a higher percentage of pts in the INO group achieved < 0.01% MRD (78.4 vs. 28.1%, *P* < 0.001). Both PFS and OS were longer with INO (median PFS, 5.0 vs. 1.8 months, *P* < 0.001; median OS, 7.7 vs. 6.7 months, *P* = 0.04)	Phase III [41]
		57 pts with R/R CD22+ B ALL received mini-hyper-CVD regimen	Mini-hyper-CVD regimen plus INO administered on day 3 of each of the first 4 cycles, rituximab (in pts whose cells were CD20-positive) and intrathecal chemotherapy were given for the first 4 courses	The ORR was 71%: 31 (53%) CR, 13 (23%) CRp, and 1 (2%) CRi. 27 (47%) pts proceeded to receive allo-HSCT. Pts who were treated with mini-hyper-CVD plus INO had a higher PFS rate and improved OS compared to a historical cohort with single-agent INO in R/R ALL (2-year PFS; 52 vs 36%; *p* = 0.20: 2-year OS; 44 vs. 25%; *p* = 0.01)	Phase II [42]
		46 pts ≥ 60 years with newly diagnosed B cell ALL. Median age is 68 years (60–81)	Mini-hyper-CVD regimen plus INO given on day 3 of each of the first 4 cycles. Rituximab (in pts whose cells were CD20-positive) and intrathecal chemotherapy were given for the first 4 courses	Of the 42 pts evaluable for response, 40 (95%) achieved CR/CRp (35 CR, 5 CRp). Of the 44 pts assessed for MRD, 41 (93%) achieved negative MRD (71% of them at CR). The mini-hyper-CVD + InO +/− rituximab (*n* = 46) results appear superior to the historical data with HCVAD +/− rituximab (*n* = 46) (3-year OS of 52 and 36%, respectively, *p* = 0.05).	Phase II [43]
CD52	Alemtuzumab	24 pts with de novo ALL in CR1. Median age was 37 years (18–77)	A target dose of 30 mg administered 3 times per week for 4 weeks (12 doses) during post-remission therapy	Of 11 pts assessed for MRD, 8 had a 1-log reduction. After 51 months of follow-up, median OS was 55 months and DFS was 53 months	Phase I [44]
		12 pts with relapsed (*n* = 11) or refractory (*n* = 1) ALL, including four relapses post-HSCT	Alemtuzumab combined with granulocyte-colony stimulating factor (G-CSF)	4 of 12 pts achieved CR, but all pts progressed within a few months and all but one died	Phase II [45]

*Pts* patients, *CIR* cumulative incidence of relapse, *OS* overall survival, *CRD* complete remission duration, *CR* complete remission, *MRD* minimal residual disease, *RFS* relapse-free survival, *DFS* disease-free survival, *R/R* refractory/relapsed, *CRp* complete remission in the absence of total platelet recovery, *CRi* complete remission with incomplete hematologic recovery, *ORR* overall response rate

**Table 2:** Common side effects of different immunotherapies

Targets	Medications	Side effects
CD20	Rituximab	Most common side effect is mild to moderate infusion reactions. Rare cases of severe mucocutaneous reactions, HBV reactivation, and progressive multifocal leukoencephalopathy
	Ofatumumab	Primarily grade 1 or 2 infusion reactions or infections
CD19	SAR3419	Dose-limiting reversible severe vision changes associated with corneal changes
	SGN-CD19A	Superficial microcystic keratopathy
	Blinatumomab	Fever, chills, and hypogammaglobulinemia are common. Serious side effects include CRS and neurotoxicity
CD22	Epratuzumab	Seizure, liver toxicity
	INO	Liver toxicity, veno-occlusive disease in transplant patients
CD52	Alemtuzumab	Severe neutropenia, CMV viremia

### CD20-targeting agents—rituximab, ofatumumab, and GA101

CD20 is a surface marker of B-lineage lymphocytes with robust expression after the mid-stage of development. It is presented in 25% of pre-B ALL and nearly all mature ALL. The GRAALL (a Group for Research in Adult Acute Lymphoblastic Leukemia) study demonstrated a higher relapse rate (39 vs 20%, *P* = 0.04) and a worse event-free survival (EFS) exclusively in pre-B ALL patients with positive CD20 expression and a high white blood count, indicating worse outcomes are associated with positive CD20 expression [6].

#### Rituximab

Rituximab is a humanized murine mAb targeting CD20. Before its introduction to ALL treatment, rituximab has shown prolonged survival for patients with CD20+ non-Hodgkin’s lymphoma and Burkitt’s lymphoma/leukemia [7]. Emerging results have confirmed its efficacy in ALL patients.

MD Anderson Cancer Center recruited patients with Ph-negative B ALL and treated with the modified hyper-CVAD (cyclophosphamide, vincristine, doxorubicin and dexamethasone) regimens with or without rituximab. For the younger (age < 60 years) subgroup, complete remission duration (CRD) and OS were better with the combination of hyper-CVAD and rituximab than with hyper-CVAD alone (69 vs 38%; *P* < .001 and 71 vs 47%, *P* = .003). However, no benefit from the addition of rituximab was noted in elderly patients (age ≥ 60 years), in part because of deaths in CR from infections. The results suggest a role for rituximab use in patients with CD20+ ALL under 60 years old [8]. Similar outcomes were observed in a multicenter randomized trial comparing the pediatric-inspired GRAALL protocol to the same regimen plus rituximab [9, 10]. Two hundred twenty patients aged 18–59 years old with newly diagnosed CD20-positive Ph-negative B cell precursor (BCP)-ALL were enrolled from 2005 to 2014. Rituximab (375 mg/m^2^) was given from induction to the first year of maintenance for a total of 16 to 18 infusions. After induction ± salvage reinduction, the CR rate was 92 and 91% in rituximab and control arm, respectively. Patients treated in the rituximab arm had a lower cumulative incidence of relapse (CIR) (2-year CIR, 18 vs 30.5% in the control arm; *p* = 0.02) and longer EFS (2-year EFS, 65 vs 52% in the control arm; *p* = 0.038), but not longer OS (2-year OS, 71 vs 64% in the control arm; *p* = 0.095). When censoring patients who received allogeneic HSCT in first CR at transplant time, EFS and OS were longer in the rituximab arm (2-year EFS, 66 vs 53%, and 2-year OS, 74 vs 63%; *p* = 0.021 and 0.018, respectively). These data indicated adding rituximab to the ALL chemotherapy protocol could improve the outcome for patients with CD20-positive, Ph-negative ALL, especially in younger adults.

Rituximab is among the most studied immunotherapies, and its safety has been well addressed. Due to its low toxicity, rituximab may be particularly useful in elder patients and patients who are unfit for more aggressive treatment [11]. However, several limitations were discovered: its efficacy is limited when administered alone; moreover, CNS relapse is common when rituximab is used as monotherapy because mAbs cannot cross the blood-brain barrier.

#### Ofatumumab

Ofatumumab (HuMax-CD20) is a second-generation anti-CD20 mAb that binds to a site different from rituximab. Ofatumumab targets a membrane proximal small-loop epitope on the CD20 molecule and is more potent than rituximab in inducing antibody-dependent cell-mediated cytotoxicity (ADCC) and complement-dependent cytotoxicity (CDC) [12]. As a single agent, ofatumumab’s adverse effects are mild, consisting primarily of grade 1 or 2 infusion reactions or infections. Only 9% of patients have grade 3 or 4 infections. In a phase II study of hyper-CVAD/MTX-Ara-C in combination with ofatumumab for adults with CD20-positive ALL, promising results have been achieved. Altogether, 55 patients with de novo ALL and 4 patients in CR with previous treatment received a median of 8 cycles (range, 1–8) of ofatumumab therapy. All but one patient (98%) achieved a CR after cycle 1, and 53 patients (93%) achieved minimal residual disease (MRD) negativity. The 3-year CRD and OS rates were 78 and 68%, respectively. The 3-year OS in patients with CD20 < 20 and ≥ 20% were 82 and 64%, respectively (*p* = 0.96). This study proved safety and high effectiveness in patients with CD20-positive ALL [13, 14]. As ofatumumab binds to a different epitope than rituximab, the medication may also be used to overcome rituximab-resistant disease. Nonetheless, longer follow-up and randomized studies are warranted in the future.

#### Obinutuzumab (GA101)

Obinutuzumab, a type II glycoengineered humanized anti-CD20 mAb, has shown potent activity in CLL and has been FDA-approved for the upfront treatment of CLL. The post-translational glycoengineering modification of obinutuzumab can enhance its binding affinity to the FccRIII receptors on immune effector cells that would promote ADCC, while at the same time decrease CDC. Obinutuzumab is potentially more potent than other anti-CD20 mAbs due to its ability to directly induce cell death [15].

Encouraging preclinical results have been reported in ALL cell lines and xenografts [12]. However, no clinical studies in ALL patients have been performed yet. Thus, future studies are awaited as it may offer another option for the treatment of ALL.

### CD19-targeting agents—SAR3419, SGN19a, and blinatumomab

CD19 is present in 90% of pre-B and mature ALL leukemic cells. Its high expression rate makes it an ideal target for immunotherapy. However, the antigen is known to internalize upon binding to antibody, making it an unsuitable target for naked mAbs. Nonetheless, the internalization property makes it an attractive target for immunoconjugate therapy.

#### SAR3419

SAR3419 is an antibody-drug conjugate (ADC) with a humanized anti-CD19 antibody conjugated to maytansin, a potent antimitotic agent. After binding to CD19-positive lymphoblast, SAR3419 is internalized and processed to release the active maytansin metabolites that induce both cell cycle arrest and apoptosis [16].

In phase I studies conducted in patients with non-Hodgkin’s lymphoma, the major dose-limiting toxicity is reversible severe blurred vision, which was associated with epithelial corneal changes. Given the adverse effect, the maximally tolerated dose was 160 mg/m^2^ intravenously every 3 weeks [17]. In studies using CD19-positive pre-B cell ALL and mixed lineage leukemia (MLL) xenografts, the administration of SAR3419 delayed disease progression, even in chemotherapy-resistant xenograft models [18]. Unfortunately, a phase II multicenter, single-arm clinical trial (NCT01440179) was prematurely terminated due to modest activity of SAR3419 compared with its competitors. In this study, patients received SAR3419 induction monotherapy (55, 70, and 90 mg/m^2^, ≤ 8 weekly dosed); the responding patients were eligible for maintenance therapy (biweekly for ≤24 weeks). Of the 17 evaluable patients, only 4 had a disease response (estimated overall response rate (ORR), 25.5%; 80% CI, 14.2–39.6%). The duration of response (DOR) was only 1.9 months (range, 1–5.6 months) [19], indicating SAR3419 monotherapy is unpromising in the treatment of R/R ALL. Thus, the unfavorable efficacy and dose-limiting toxicity may hinder further development of SAR3419.

#### SGN-CD19A

SGN-CD19A (denintuzumab) is a novel ADC composed of a humanized anti-CD19 mAb linked to a microtubule-disrupting agent monomethyla-uristatin F (MMAF). The ADC binds to CD19, internalizes, and releases MMAF, which ultimately results in G2-M phase growth arrest and induction of apoptosis. In a phase I dose-escalation study (NCT 01786096), 49 patients with R/R ALL (*n* = 40) or lymphoma (*n* = 9) were treated with weekly IV SGN-CD19A (0.3–4.5 mg/kg) or every 3 weeks (0.5–6 mg/kg). Among the 33 evaluable patients, objective responses were observed in 30% (10 of 33), including 6 of 25 patients on the weekly schedule and 4 of 8 patients on the every 3-week schedule. SGN-CD19A was generally well tolerated with superficial microcystic keratopathy being the most common toxicity, which might require routine steroid eye drop prophylaxis [20]. After all, promising results in heavily pretreated R/R patients and safety profile suggest opportunities for combination with other conventional anti-leukemic therapies in lymphoblastic malignancies.

#### Blinatumomab

Blinatumomab is the first-in-class BiTE construct, which binds to both CD3 on CTLs and CD19 on B cells. The construct can facilitate CTL activation and expansion, which will result in effective lysis of CD19-positive cells, through release of cytokines and the pore-forming perforin system [21, 22]. (Fig. 1).

Pharmacokinetic analyses from a phase II study in ALL patients found rapid and sustained depletion of B cells, with the level becoming undetectable at a mean of 2.18 days (range, 0.03 to 13.94 days) [23, 24]. On the other hand, circulating T cells were initially depleted from the peripheral blood within hours of blinatumomab infusion but recovered to baseline or higher within a few days, followed by a polyclonal expansion of T cells expressing the activation marker CD69 [23]. Accordingly, many patients experienced a CRS 1–2 days after the infusion, which was mediated by transient release of inflammatory cytokines interleukin (IL)-2, IL-6, IL-10, tumor necrosis factor and interferon-γ from blinatumomab-engaged T cell effectors. Cytokine levels declined after day 2, and these spikes generally did not recur with future cycles [25].

In a phase II study of 21 patients with persistent MRD, the single-agent blinatumomab was continuously infused at 15 μg/m^2^/day for a 4-week period followed by a 2-week treatment-free interval before starting the next cycle. The study achieved a molecular response rate of 80% and long-term relapse-free survival (RFS) of 61%, after a median follow-up of 33 months [24]. In the BLAST study, 88 of the 106 ALL patients (76%) achieved MRD after the first cycle of blinatumomab, including high-risk subgroups such as patients in second-line treatment, patients with high MRD burden, and older patients. With median follow-up of 29.5 months, median OS and RFS were 18.9 and 36.5 months, respectively. MRD complete response achieved in the first cycle was associated with longer OS (median OS 40.4 vs 12.0 months, *P* = .001) and RFS (35.2 vs 7.1 months, *P* = 0.002), compared with not achieving an MRD complete response after the first cycle [26].

Based on the positive experience in adult patients with MRD-positive B cell lineage ALL, a phase II study of blinatumomab enrolled 36 Ph-negative pre-B ALL adults with primary refractory disease or relapsed after chemotherapy or HSCT. It led to 69% CR or CR with incomplete count recovery (CRi), and 88% of the responders also obtaining a molecular response (MRD level below 10^− 4^ by PCR) within the first two cycles of drug administration. The median OS was 9.8 months (95% CI, 8.5 to 14.9 months), and the median RFS was 7.6 months (95% CI, 4.5 to 9.5 months) [27]. Another study of 189 patients with high-burden R/R B ALL showed a CR/CRi rate of 43% and a median OS rate of 6.1 months [28]. Safety profile appears favorable in these studies, with CRS and neurologic events being the most severe toxicities.

Despite the promising initial results, some patients do not respond to blinatumomab or experience disease progression after an initial response. The causes of primary resistance remain unknown. However, several mechanisms have been proposed. One possible mechanism is the selection of CD19-negative subclones, as leukemic cells can still maintain proliferation without CD19 expression [29]. Further, increased regulatory T (Treg) cells in combination with high lactic dehydrogenase level predicted resistance to blinatumomab [30]. Activated Tregs secrete IL-10 suppressing T cell proliferation, leading to treatment failure. Upregulation of programmed death-ligand 1 (PD-1) on leukemia cells after blinatumomab treatment is also a potential immune escape mechanism [31, 32]. Further studies are warranted to analyze the significance of the PD-1/PD-L1 interplay as a resistance mechanism to blinatumomab.

Besides blinatumomab, other BiTEs are also under active developing. Recently, a disulfide-stabilized form of BiTE was tested which showed increased efficient [33].

### CD22-targeting agents—epratuzumab and inotuzumab ozogamicin

CD22 is expressed on leukemic blasts in 90% of pre-B ALL and mature ALL. Like CD19, CD22 is rapidly internalized upon antibody binding. Possible mechanisms of action of anti-CD22 antibodies include ADC, modulation of B cell signaling, and inhibition of proliferation [34].

#### Epratuzumab

Epratuzumab is a naked, humanized anti-CD22 immunoglobulin G1 (IgG1) that is internalized after binding to CD22. Engagement of CD22 with epratuzumab leads to direct phosphorylation of key upstream inhibitory receptors of BCR signaling [35]. Epratuzumab has been used as a single agent (4 doses of intravenous epratuzumab 360 mg/m^2^ twice a week) followed by standard Children’s Oncology Group (COG) reinduction chemotherapy regimen in 15 pediatric patients with relapsed pre-B ALL. Although this strategy did not improve CR in comparison with historical controls (65 vs. 66%), more patients achieved negative MRD status (42 vs. 25%, *p* = 0.001). The more favorable rate of MRD negativity after addition of epratuzumab suggests that this antibody may enhance the response to cytotoxic chemotherapy [36]. However, further follow-up is needed to determine whether the deeper remission level translates into improved DFS or OS. In adult ALL, the Southwest Oncology Group (SWOG) evaluated epratuzumab combined with clofarabine plus cytarabine in 31 patients experiencing first or later relapsed disease. Overall, the response rate was 52%, significantly higher than that of SWOG’s previous trial with clofarabine/cytarabine alone (17%) [37]. These data provided the groundwork for a randomized phase III trial in children with relapsed ALL evaluating chemotherapy with or without epratuzumab (NCT01802814). Recently, in a phase II prospective study, epratuzumab was combined with hyper-CVAD in younger patients (18–59 years old) with R/R CD22+ B ALL. Among the 30 patients ultimately considered for analyses, the ORR was only 50%, with 45% of CR/CRi patients achieved negative MRD. All patients in CR/CRi and 1 patient in PR received a consolidation chemotherapy. However, at the time of analysis, all patients died except for the recently enrolled responders still in CR [38]. The short-lived improvement and overall disappointing outcome could be explained by an insufficient disease load decrease and/or by escape of the blast cells to epratuzumab. The results suggested that epratuzumab should be tested within first-line chemotherapies as it may participate to decrease the MRD level. Because CD22 is internalized upon binding, we expect conjugating cytotoxic agents with epratuzumab would result in more favorable outcomes.

#### Inotuzumab ozogamicin (INO)

Inotuzumab ozogamicin (INO) is an ADC that consists of calicheamicin (a potent DNA-binding cytotoxic agent) attached to an engineered humanized monoclonal immunoglobulin G4 (IgG4) antibody targeting CD22. INO binds CD22 with sub-nanomolar affinity and is rapidly internalized, delivering the conjugated calicheamicin intracellularly. Calicheamicin binds to the minor DNA grove, causing double-strand DNA cleavage and cell apoptosis.

In a single-institution phase II study, 49 patients with R/R ALL were treated with 1.8 mg/m^2^ INO every 3–4 weeks as a single agent. It resulted in an ORR of 57% (18% CR and 39% CRi) and median OS of 5.1 and 7.9 months in all patients and 28 responders, respectively. A total of 27 patients who achieved complete morphological response were assessed for MRD, and 63% (17 of 27) of patients was found to have MRD-negative status. Abnormal liver function tests were the most significant adverse events observed and were graded as severe in 31% of patients [39]. To optimize the benefit-risk ratio of INO, a weekly dosing regimen was evaluated in 41 patients based on preclinical studies [40]. Weekly drug administration at a lower dose (0.8 mg/m^2^ on day 1, 0.5 mg/m^2^ on days 8 and 15) was associated with similar efficacy (59% ORR). However, liver toxicity was seen in only 11 of 41 (27%) patients with weekly INO compared with 28 of 49 (57%) with single-dose INO. This study indicates that more frequent but lower dose of INO may reduce toxicities while maintain the efficacy. Recently, a global, open-label, randomized phase III study confirmed the notion. Patients were randomly assigned to receive either INO (0.8–0.5 mg/m^2^, weekly, three times per cycle, cycle length of 21–28 days, 6 total cycles) or standard intensive chemotherapy. The CR rate was higher with INO than with standard chemotherapy (80.7 vs. 29.4% *p* < 0.001). Moreover, a higher percentage of patients in the INO group achieved MRD-negative status (78.4 vs. 28.1%, *P* < 0.001). Both PFS and OS were longer with INO (median PFS, 5.0 vs. 1.8 months, *P* < 0.001; median OS, 7.7 vs. 6.7 months, *P* = 0.04) [41]. Because HSCT is considered to be the only curative treatment option, the capacity of INO to increase the number of patients who can proceed to HSCT is encouraging.

The promising results of INO as a single agent led to the initiation of a clinical trial combining INO with non-myelosuppressive chemotherapy. A total of 57 patients with R/R CD22+ B ALL received INO, in combination with mini-hyper-CVD regimen, rituximab, and intrathecal chemotherapy. The ORR was 71%, with 47% patients proceeding to allo-HSCT. Compared with historical cohort, patients treated with mini-hyper-CVD plus INO had a higher PFS rates and improved OS (2-year PFS; 52 vs. 36%; *p* = 0.20: 2-year OS; 44 vs. 25%; *p* = 0.01) [42]. These encouraging results provided an option of combining INO with low-intensity chemotherapy in patients with R/R ALL.

The same regimen of mini-hyper-CVD combined with INO was also evaluated in elderly patients ≥ 60 years with newly diagnosed B ALL [42]. Of the 42 patients evaluable for response, 40 (95%) achieved CR/CRp. Of the 44 patients assessed for MRD, 41 (93%) achieved negative MRD. The results appear superior to historical data with hyper-CVAD alone in a similar patients’ population (3-year overall survival (OS) rates of 52 and 36%, respectively, *p* = 0.05) [43].

### CD52-targeting agent—alemtuzumab

CD52, an antigen involved in T cell activation, is expressed in 70% of T ALL and pre-B ALL. Alemtuzumab is a fully humanized monoclonal antibody against CD52. Alemtuzumab has been evaluated in the CALGB phase I trial of 24 patients with de novo CD52+ ALL after the successful achievement of CR with induction chemotherapy [44]. The drug was given three times weekly for a target dose of 30 mg subcutaneously. After 51 months of follow-up, median OS was 55 months and DFS was 53 months. Notably, alemtuzumab was associated with increased risk of neutropenia and CMV viremia, supporting a role for stimulating factor support. Recently, a phase II study assessed the efficacy of alemtuzumab combined with granulocyte colony-stimulating factor (G-CSF) in 12 patients with R/R ALL. G-CSF was administered during alemtuzumab administration. Although 4 of the 12 patients achieved CR, all patients progressed within a few months and all but 1 died [45]. The current studies only demonstrate modest activity of alemtuzumab against T ALL or B ALL, yet significant adverse effects have been reported extensively. These results would potentially limit further development of alemtuzumab.

### Chimeric antigen receptor T cell therapy

Chimeric antigen receptors (CARs) consist of an extracellular binding domain (scFv), hinge domain, transmembrane domain, and intracellular signaling domains. When expressed in autologous T cells (or donor T cells in the post-transplant setting), CARs redirect the CTLs toward antigen-expressing tumor cells in an HLA-independent manner. DNA constructs encoding such CARs could be stably incorporated into human T cells via lentiviral or γ-retroviral transductions, electroporation, or “Sleeping Beauty” transposon.

The engineering of CAR T cells evolves over time. The first-generation CAR T cells are only incorporated with T cell receptor CD3ζ signaling domain. This construct revealed weak proliferation ability, poor anti-tumor effect, and short survival of T cells [46, 47]. Second- and third-generation CAR T cells are incorporated with costimulatory domains, such as CD137 (4-1BB), CD28, CD27, or CD134 (OX40), which significantly enhanced the expansion, persistence, and potency of CAR T cells. Among them, CD28 and 4-1BB are currently the most widely used costimulatory domains. Studies showed that CD28 endued CAR T cells with stronger killing ability, while 4-1BB granted longer persistence in vivo.

Expansion of CAR T cells is essential. The CAR T cells are first expanded ex vivo to a goal cell count around 3 × 10^6^ cells/kg [48, 49]. Following infusion, circulating CAR T cells expand in vivo by 1000 folds within 7–14 days. CAR T cells have the potential to enter cerebrospinal fluid, exerting anti-leukemic activity in this sanctuary site, and persist after infusion [50]. However, the persistence of CAR T cells within patients varied across the studies. Stephan et al. report persistence of CD19-CAR T cells for 3–39 months after infusion in patients with ongoing responses, whereas Davila et al. found the levels undetectable by 3 months [51, 52].

#### Clinical outcome of CD19-targeted CAR T cells in R/R ALL

CAR T cell therapy has achieved promising results in multiple clinical trials (Table 3). The majority of the trials are led by three research institutes—the University of Pennsylvania (UPenn), the National Cancer Institute (NCI), and the Memorial Sloan Kettering Cancer Center (MSKCC).

**Table 3:** Major clinical trials on anti-CD19 CAR T cell therapy

Institution and trial no.	Costimulatory domain and gene transfer	Patient	Lymphodepleting chemotherapy	Cell dose	Outcomes
MSKCC NCT01044069 [53]	CD28, γ-retrovirus	Adults with R/R ALL including Ph + ALL, *n* = 44	Cy or flu/cy	1–3 × 10^6^ cells/kg	CR: 36/43 (84%) with 29/35 (83%) of responders negative for MRD; OS: 76% (MRD-CR cohort) and 14% (MRD + CR cohort) at 6 months
NCI NCT01593696 [54]	CD28, γ-retrovirus	Children and young adults with R/R ALL, *n* = 51	Cy, low-dose flu/cy, FLAG, ifosfamide/etoposide or high-dose flu/cy	0.03 × 10^6^–3 × 10^6^ cells/kg	CR: 31/51 (60.8%) with 28/31 (90%) of responders negative for MRD OS: 34.7% (receiving flu/cy) at 38 months LFS: 49.5% (MRD- CR) at 18 months LFS: 62% (MRD- CR cohort having a subsequent HSCT) at 18 months
Multicenter studies NCT02614066, NCT02625480 [55]	CD28, γ-retrovirus	R/R ALL aged ≥ 18 years (ZUMA-3) or 2–21 years (ZUMA-4) with ≥ 25% marrow blasts Ph+ ALL and low-burden central nervous system disease are eligible	Flu/cy	1 or 2 × 10^6^ anti-CD19 CAR T cells/kg	CR: 5/5 (100%)
FHCRC NCT01865617 [72]	4-1BB, *Lentivirus*	Adult with R/R ALL, *n* = 30 29 evaluable	Cy ± etoposide or cy/flu	2 × 10^5^ or 2 × 10^6^ or 2 × 10^7^ cells/kg 1:1 CD4+:CD8+	CR: 27/29 (93%)
UPenn/CHOP NCT01626495 [56]	4-1BB, *Lentivirus*	Children and young adults with R/R ALL, *n* = 53	Investigator’s choice	1–17.4 × 10^6^ cells/kg	CR: 50/53 (94%) with 47/50 (94%) of responders negative for MRD OS: 78% at 12 months RFS: 72% at 6 months
ELIANA (global trial) NCT02435849 [57]	4-1BB, *Lentivirus*	Children and young adults with R/R ALL, 29 pts reaching D28 prior to the data cutoff	Flu/cy	0.2–4 × 10^6^ cells/kg	CR: 24/29 (83%) with all of responders negative for MRD
ENSIGN (US multicenter trial) NCT02228096 [58]	4-1BB, *Lentivirus*	Children and young adults with R/R ALL, *n* = 29	Flu/cy, or none due to leukopenia	2–5 × 10^6^ cells/kg for ≤ 50 kg, 1–2.5 × 10^8^ cells for > 50 kg	ORR (CR + CRi): 20/29 (69.0%) with 18/29 (62.1%) responders negative for MRD RFS: 66.4% at 6 months OS: 75.7% at 6 months
UPenn/CHOP NCT02374333 [74]	4-1BB, *Lentivirus* humanized anti-CD19 scFv domains	Children and young adults with R/R ALL, with or without prior exposure to a CAR T cell product, *n* = 30	Flu/cy	No mention	CR: 26/30 (87%) CR for patients previously treated with CAR T: 7/11(64%) with 5/7 (71%) of responders negative for MRD CR for patients with no prior exposure to CAR T: 19/19 (100%) with 19/19 (100%) of responders negative for MRD

*MSKCC* Memorial Sloan Kettering Cancer Center, *NCI* National Cancer Institute, *FHCRC* Fred Hutchinson Cancer Research Center, *UPenn* University of Pennsylvania, *CHOP* Children’s Hospital of Philadelphia, *Cy* cyclophosphamide, *Flu* fludarabine, *FLAG* fludarabine, high-dose cytarabine, and G-CSF

A recent study from MSKCC reported the efficacy of CAR T cell therapy in 44 adults with R/R B ALL. Of the 43 patients evaluable for response, 36 patients (84%) achieved CR after 19-28z CAR T cell (JCAR015, Juno Therapeutics, Seattle, WA) infusion. Among them, 29 (83%) achieved MRD negativity (MRD-CR). The median OS for all patients and MRD-CR patients was 8.5 and 10.8 months, respectively. Posttreatment MRD status emerged as a strong predictive marker of OS. The OS at 6 months was 76% (95% CI 51–89) in the MRD-CR cohort vs. 14% (95% CI 8–45) in the MRD+ CR cohort. However, this study did not show significant survival superiority in patients who underwent allo-HSCT after CAR T cell infusion than those who did not (OS at 6 months was 70% in patients who underwent allo-HSCT vs. 64% in patients who did not receive allo-HSCT) [53]. In contrast, researchers from NCI showed that long-term outcomes were superior among patients with post-CAR T HSCT. In their study, 31 (60.8%) of 51 R/R ALL patients achieved a CR with 28/31 (90%) of responders negative for MRD. Relapse was significantly more common in patients who did not have a HSCT after CAR T therapy (6/7; 85.7%) compared to those who did (2/21; 9.5%) (*p* = 0.0001). Even when counting in the transplant-related mortality, the median leukemia-free survival (LFS) in the HSCT group was significantly longer (HR = 16.9, *p* = 0.0006) [54]. Based on the aforementioned excellent outcomes, a multicenter, phase 1/2 study called ZUMA-3 and ZUMA-4 that enroll adult (aged ≥ 18 years) and pediatric (aged 2–21 years) R/R ALL patients were started [55]. Researchers from the UPenn also reported their experience with CTL019 (Novartis, Basel, Switzerland) in patients with R/R ALL. In a recent study of largely pediatric patients, 94% (50/53) achieved CR, with MRD negativity in 47 responding patients. RFS was 72% at 6 months (95% CI, 59–87%) and 44% at 12 months (95% CI, 30–65%), and OS was 78% at 12 months (95% CI, 67–91%) [56]. This single-center trial of CTL019 for R/R ALL showed prolonged CAR T cell persistence along with long-term CR without further therapy in the majority of patients. However, the high efficacy and a similar safety profile of CTL019 have yet to be reproduced in multicenter trials, such as the first US multicenter trial (ENSIGN) and the first global trial (ELIANA) [57, 58].

Although the above studies differed in CAR designs, T cell manufacturing, conditioning regimens, patients’ age, leukemia burdens, and T cell dosages, each trial was comparably effective in treating R/R ALL, reaching 90% even in heavily pretreated patients. As a result, these preliminary clinical trials have paved the way for more ongoing studies.

In addition to autologous CD19-CAR T cells, the potential of donor-derived CD19-CAR T cells to treat relapse after allo-HSCT was also intriguing [59]. Some preliminary results have been reported [60, 61]. Brudno et al. conducted a clinical trial using allogeneic donor-derived CAR T cells in B cell malignancy patients who relapsed after allo-HSCT [62]. Eight of the 20 treated patients obtained remission. None of the patients developed new onset graft-versus-host disease (GVHD) after the infusion. The result was similar with a previous study from Baylor College of Medicine using donor-derived CD19-redirected virus-specific T cells, in which no evidence of GVHD was reported [61]. However, in another study, Dai et al. reported for the first time of GVHD occurrence in 2 of the 3 patients that received donor-derived CAR T cells [63]. The GVHD might be related to a higher dose of infused CAR T as well as mixed chimerism in recipients. In addition, different CAR designs may also play a role. Using a mouse allo-HSCT model, Smith et al. found that adoptive transfer of murine donor m1928z CAR T cells (CAR with CD28 costimulatory domain) caused significantly less GVHD compared to m19delta T cells (CAR with no costimulation) and m19BBz T cells (CAR with 4-1BB costimulatory domain) [64]. This result is in accordance with the clinical observations, as the 4-1BB domain was incorporated in the donor-derived CAR T cells that caused GVHD in Dai’s case [63].

Even more interestingly, allogeneic CD19 CAR T cells could be applied to patients without prior HSCT. The infusion of allogeneic CAR T cells could potentially augment the killing effect through additional alloreactive-attacking capability. Cai et al. reported a case where the patient received co-infusion of haplo-identical allogeneic CAR T cells and mobilized peripheral blood stem cells (PBSCs) following induction therapy. The patients achieved MRD-CR and full donor engraftment, with only mild toxicity [65]. Although the result seems intriguing, potential complications such as GVHD need further study.

#### Toxicities of CD19-targeted CAR T cells

Toxicities associated with CAR T cell therapy are reported in nearly all clinical trials. Most toxicities were mild and reversible, including fever, chills, hypotension, dyspnea, headaches, and fatigue. The three main concerning toxicities are on-tumor off-target toxicity, CRS, and neurotoxicity. On-tumor off-target toxicity is related to the inability of CAR T to distinguish between tumor and normal cells, since the CD19 antigen is homogeneously expressed by both normal and malignant B lymphocytes. The toxicity can lead to B cell aplasia, which might cause agammaglobulinemia. Low immunoglobulin level will increase the risk of opportunistic infections. But it is preventable with immunoglobulin infusions and/or administration of antibiotics. CRS is caused by significant production of inflammatory cytokines secreted by activated CAR T cells [66, 67]. The onset of CRS is variable, ranging from 24 h to 3 weeks following CAR T infusion [51, 68]. While mild CRS is reported in almost all patients, severe CRS occurs in about 30% of patients. Patients with high leukemia burden are at increased risk of more severe CRS, presenting as vascular leak and hypotension leading to multi-organ system dysfunction. These severe complications will often need aggressive medical management, including hemodynamic support, mechanical ventilation, and application of tocilizumab (an IL-6 inhibitor) and, in life-threatening conditions, corticosteroids. It is worth noting, however, that administration of corticosteroids may also minimize or eliminate the CAR T cell activity. Neurological toxicities are also accompanied by CAR T infusion, including delirium, dysphasia, akinetic mutism, and seizures. In November 2016, two patients died from cerebral edema in a phase II ROCKET study testing the investigational anti-CD19 CAR T cell JCAR015 (Juno Therapeutics, Seattle, WA). Considering in the previous three deaths in July 2016, the trial was halted due to concern of safety [69]. Juno highlighted the lower levels of toxicity in both ALL and other B cell malignancies using their 4-1BB CAR T products with defined CD4:CD8 composition (JCAR017 and JCAR014). Thus, they speculated the flu/cy conditioning regimen or the CD28 costimulatory domain used in JCAR015 that may trigger more rapid expansion of T cells and disruption of the blood-brain barrier might be the culprits. However, other groups (such as Kite/NCI) using CD28 CARs appeared to have no significant neurotoxicity, even though they also used flu/cy for conditioning. Thus, the etiology remains unproven.

#### Causes and management of relapse after CAR T therapy

If CAR T could be durably detected in the recipients, the chances of relapse would decrease [70]. In one study, loss of functional persistence of CAR T was associated with CD19+ relapse with a hazard ratio (HR) of 34 (*p* = 0.013) [71]. Some studies sought to prolong CAR T persistence by reinfusion of CAR T in patients with evidence of poor persistence. However, investigators from Fred Hutchinson Cancer Research Center (FHCRC) detected a cytotoxic CD8+ T cell response to CAR in some patients who failed to achieve engraftment of CAR T cells after second infusions; epitope mapping in 1 patient identified immunogenic epitopes within the murine FMC63 scFv [72]. Thus, repeated infusion would be ineffective in this subset of patients with rapid CAR cell loss mediated by immune rejection. In this condition, retreatment with CD19-directed CAR T expressing a humanized scFv may induce second remission of ALL [73]. UPenn has published the results of a phase I study of humanized CTL119 at the 2016 ASH meeting. Thirty children and young adults with R/R B ALL with or without prior exposure to CAR T cells were enrolled. They found CTL119-induced remissions in 64% (7/11) of patients previously treated with murine CD19-directed CAR T cells and 100% of CAR-naïve patients [74]. Inadequate lymphodepletion may be another reason for limited CAR T persistence. Indeed, addition of fludarabine to the lymphodepletion regimen was showed to enhance CAR T persistence and prevent transgene rejection [75]. In addition, combining CAR T therapy with other medications such as PD-1 inhibitor [76] or BTK inhibitor [77] was also showed to improve CAR T cell persistence. Loss of surface CD19 expression is another cause of relapse [69]. Interestingly, Lacey et al. reported a case where the relapsed CD19-negative leukemia was originated from a single leukemic cell accidentally transduced with CAR19 that survived the manufacturing process. This leukemic clone evaded CTL019 detection via downregulation of the target antigen in a cell autonomous fashion [78]. Recently, some promising studies investigated the use of CAR T targeting CD22 to treat CD19-negative relapses [79–81]. Targeting additional B cell antigens is an active area of investigation.

## Conclusions

With the rapid development of naked monoclonal antibodies, antibody-drug conjugates, bispecific T cell engagers, and adoptive T cell therapies, the era of R/R ALL treatment is fast forwarding. CD19, CD20, CD22, and CD52 are the main markers presented in the majority of B ALL patients and thus are targeted in immunotherapies. With CAR T therapy moving to the market, achieving higher CR rates than frontline chemotherapies or even chemotherapies combined with mAbs, the roles of mAbs need to be newly defined. Monoclonal antibodies may still play important roles in induction therapy of ALL due to their availability and relatively safe profile. In the future, immunotherapies may substitute or allow a lower dose of chemotherapy to achieve long-term remission in ALL patients, even in those with poor performance status who are otherwise ineligible for traditional chemotherapy. Despite high remission rate that could be achieved by the aforementioned therapies, relapse still remains as the major problem. As the choices of targeted and immune therapies increase, we must look beyond high remission rates and search for the strategies to prevent relapse and reduce toxicities.

## Abbreviations

ADC: Antibody-drug conjugate
ADCC: Antibody-dependent cell-mediated cytotoxicity
ALL: Acute lymphoblastic leukemia
BiTE: Bispecific T cell engager
CAR: Chimeric antigen receptor
CDC: Complement-dependent cytotoxicity
CR: Complete remission
CRi: Complete remission with incomplete blood cell count recovery
CRp: Complete remission with incomplete platelet recovery
CRS: Cytokine release syndrome
EFS: Event-free survival
HR: Hazard ratio
HSCT: Hematopoietic stem cell transplantation
INO: Inotuzumab ozogamicin
mAb: Monoclonal antibody
MRD: Minimal residual disease
ORR: Overall response rate
OS: Overall survival
R/R: Refractory/relapsed
RFS: Relapse-free survival
